# Detecting temporal clinical change in sequential chest radiography reports: a multi-model and multi-prompt evaluation of large language models

**DOI:** 10.3389/fdgth.2026.1919939

**Published:** 2026-09-04

**Authors:** Ertürk Erdağı

**Affiliations:** Department of Computer Engineering, Istanbul Medeniyet University, İstanbul, Türkiye

**Keywords:** chest radiography, clinical natural language processing, large language models, prompt engineering, radiology reports, temporal change detection, temporal reasoning

## Abstract

**Background:**

Radiology reports contain critical information for monitoring patients' clinical progress and evaluating treatment outcomes. Sequential radiological images are particularly valuable, providing not only current findings but also comparative assessments against previous reports. Automatically detecting temporal changes between free-text reports remains a significant challenge in Natural Language Processing.

**Methods:**

This study evaluated the performance of Large Language Models in detecting temporal novelty using the LUNGUAGE dataset, which contains sequential chest radiography reports. The comparative multi-model and multi-prompt evaluation was carried out on a fixed, class-stratified subset of 1,500 of these examples. Seven language models from OpenAI, Anthropic, and Google Gemini were tested across five prompt strategies: zero-shot, few-shot, structured reasoning, memory-augmented, and temporal graph prompting. Each model classified target findings as new, improved, worsened, stable, or resolved by evaluating current and previous reports together. Performance was measured using accuracy, macro-F1, weighted-F1, and class-based metrics. The “new” class was analyzed separately due to its clinical importance. Model errors were categorized into clinically interpretable types, including missed new findings, missed resolved findings, temporal errors, and change direction errors. Bootstrap confidence intervals and paired McNemar significance tests assessed performance uncertainty.

**Results:**

GPT-4.1 emerged as the top-performing model. Its few-shot prompt strategy achieved the best results: 0.8369 accuracy, 0.7888 macro-F1, 0.8322 weighted-F1, and 0.5385 recall for the “new” class.

**Conclusion:**

These findings demonstrate that Large Language Models hold substantial promise for temporal clinical reasoning in sequential radiology reports, and that performance depends not only on model architecture but also on prompt strategy and the class being evaluated.

## 1 Introduction

Radiology reports contain written data of visual findings that are part of clinical decision-making processes. Chest radiograph reports may include significant changes such as the appearance, persistence, regression, worsening, or disappearance of findings such as pneumonia, pleural effusion, atelectasis, consolidation, pulmonary edema, cardiomegaly, and similar conditions. In clinical practice, more important than whether a finding is included in the current report is observing how it has changed compared with previous reports. In radiology, this is important for comparing successive radiology reports, evaluating disease status, monitoring treatment response, planning follow-up, and prioritization.

Radiology reports are written in free-text format. The same finding can be phrased differently across reports, and comparisons with prior studies are often implicit; in this case, keyword matching or report classification is insufficient to capture temporal change.

Clinical Natural Language Processing (NLP) has made significant progress in tasks such as finding extraction, entity recognition, report classification, clinical coding, and summarization in reports. Large-scale datasets such as MIMIC-CXR have made radiology reports in free text format related to chest radiography available to researchers, and studies in this field have accelerated (1). Large Language Models (LLMs) in healthcare have been an important tool in tasks such as text classification, information extraction, summarization, answering clinical questions, and decision support systems (2–6). The use of LLMs in radiology is increasing in tasks such as extracting information from radiology reports and interpreting impressions from radiological findings (7, 8).

Temporal reasoning is a fundamental problem in medicine, relevant to diagnosis, decision support, and temporal abstraction (9). LLM-based clinical temporal relation extraction generally builds event-time or patient-time timelines, and can be improved with prompt design.

Most existing work operates at the single-report level, and studies that decide, at the finding level, whether a finding is new, stable, improving, worsening, or resolved between a patient's consecutive reports remain limited; temporal-relation methods rarely convert the extracted relations into a clinical decision label. Unlike temporal-relation extraction, which links event and time expressions within a text, temporal novelty detection makes a clinical comparison between two separate reports: it requires matching clinical entities across reports, checking whether a finding existed previously, and determining whether the current finding is new.

This study has many original contributions. These contributions are:

- Temporal novelty detection in consecutive chest radiography reports was performed at the findings level, and this process was treated as a clinically significant evaluation task.
- Different models from the OpenAI, Anthropic Claude, and Google Gemini providers were compared using the same data and evaluation methodology with multiple prompt strategies.
- In this study, the evaluation was not limited to general criteria such as accuracy and F1-score, but also included clinically significant criteria such as macro-F1 and “new” class recall.
- Model errors were categorized into clinically interpretable temporal error types, analyzing situations where new findings were missed, the direction of change was not captured, or resolved findings were not interpreted correctly.
- The statistical uncertainty of model-prompt comparisons was analyzed using bootstrap confidence intervals.

## 2 Literature review

LLMs are increasingly used in healthcare for processing free text, extracting information, summarizing reports, communicating with patients, and providing clinical decision support. Studies in the healthcare show that these models offer high language comprehension capacity in clinical texts, but research is needed on issues of hallucination, reliability, explainability, and domain-specific evaluation (3, 10). This suggests that LLMs should be considered not as direct clinical decision-makers, but as auxiliary systems with a controlled and task-specific evaluation structure. This study focuses on detecting the temporal change of findings on consecutive reports, rather than on a general healthcare text production problem.

In the field of health, LLM-based research has gained prominence in recent years through approaches at the task-specific, field-specific, and clinical context levels. The LLM structure created for mental health triage can be used as a decision support system by processing long and variable clinical records (11). One of the field-specific structures is the OphGLM model, which is language-vision-based. This model was developed in the field of ophthalmology and shows that visual and textual information can be modeled together (12). These studies have shown that the success of LLMs in the medical field depends on their general language skills as well as its modeling in accordance with the task structure.

Radiology reports are important in the field of clinical NLP. Reports are generally written in free text. Therefore, NLP techniques are needed for tasks such as information extraction, clinical coding, report summarization, and explanation generation. There are studies conducted using LLMs for information extraction from radiology reports. A large part of these studies enable the generation of structured clinical information using free text reports (7). These studies show that the texts in radiology reports are suitable for LLM-based systems. However, most of these studies focus on information extraction at the individual report level. Studies on whether the finding is new, stable, improving, or worsening among consecutive reports of the same patient, which is the main focus of this study, are limited.

LLM performance in extracting information from radiology reports is an important research topic. Studies demonstrating high performance in extracting information from radiology reports in free text format show that LLMs are effective in radiology report structuring tasks without requiring retraining (13, 14). The information extraction task usually involves measuring or clinically expressing a finding in the report. The task addressed in this study is at a more complex level. In this study, the model not only recognizes the finding but also compares it with the information in the previous report and determines the temporal context. Therefore, while studies on information extraction form the basis of this study, they are not sufficient on their own in terms of comparison between consecutive reports.

Summarizing radiology reports and generating impressions is important for LLMs. Studies are comparing the performance of zero-shot and few-shot strategies in prompt-based assessments for summarizing findings in radiology reports with LLMs. These studies emphasize the need for clinical expert evaluation along with automated metrics (15). Studies aimed at generating impressions from report findings transform the texts in radiology reports into a more clinically usable form for LLMs (16). However, summarizing aims to rephrase the information in the report. Temporal novelty detection requires the identification of changes between two reports. In this respect, this study transforms the prompt-based assessment strategy in report summarizing into a more complex temporal reasoning problem.

Current studies on radiology reports are characterized by information-driven and self-refining reasoning. Self-critical multi-source information frameworks are one such approach. These frameworks combine structured clinical information, visual evidence, and iterative self-improvement within a single LLM architecture. This approach goes beyond linear generation and adds reasoning and self-evaluation to the process (17). A similar approach is memory-based visual-language converters. These models capture long dependencies and combine visual and textual features. This allows for better handling of underrepresented findings in unbalanced datasets (18). All of these approaches aim to generate complete reports from images. The performance of this generation is evaluated using metrics such as BLEU and METEOR. This study has a different objective. Sequential reports found in the dataset and written are taken as input, and the target finding among the reports is classified temporally. The evaluation process is performed using clinical classification metrics and error taxonomy.

In radiology, LLMs need to be implemented in a safe and standardized manner. Simplifying radiology reports, structuring report content, and generating explanations for patients show that the model should be evaluated not only in terms of accuracy but also in terms of clinical suitability and error type (19). This situation is directly related to the evaluation structure of the study. Because in this study, models were not only evaluated with general metrics, but also with metrics such as macro-F1, new class recall, error type distribution, and bootstrap confidence intervals. In this way, the success of the models was not evaluated with a single score, but a comprehensive evaluation was presented by considering clinical risk and statistical uncertainty.

Temporal information extraction in clinical texts is an important research topic. Clinical reports and electronic health records contain time-dependent information such as disease course, treatment response, symptom onset, and finding change. Clinical temporal relation extraction studies model the relationships between clinical events and time expressions and create patient timelines. Temporal relation extraction studies on pediatric clinical reports strongly reveal the clinical time relationships of LLMs (20). In this study, the time relationship is transformed into a clinical decision. Determining the status of the radiological finding in relation to the previous report forms the basis of this decision-making study.

Recent studies in radiology show that model evaluation progresses in the form of more reasoning, structured data, and clinical context. Studies developed to evaluate diagnostic reasoning in chest radiographs show that it is not enough to simply classify correctly; the model's decision-making process must be clinically meaningful (21). A similar process was carried out in this study, but instead of image-based work, text-based work was performed on reports at the temporal level. This situation can be positioned as complementary between image-centric radiology artificial intelligence studies and text-centric clinical NLP studies.

A holistic evaluation of the studies found in the literature reveals that LLM-supported studies in the healthcare field mostly focus on reliability and clinical application studies. At this point, it has been observed that studies involving multiple models and prompts on the temporal reasoning task in radiology remain limited. Studies on radiology reports generally focus on extracting information from single reports and summarizing reports; however, studies examining temporal changes at the finding level between current and previous reports are limited. Clinical temporal relation extraction studies can reveal temporal relationships, but their conversion into clinical decision labels is limited. This study addresses these research gaps using an open-access dataset and a multiple-model and multiple-prompt configuration. The study was comprehensively evaluated using Google, OpenAI, and Anthropic model providers and different evaluation metrics. This configuration, using LLMs, revealed not only the level of accurate prediction but also the error level and ambiguity in clinical temporal situations in consecutive radiology reports.

## 3 Materials and methods

The main objective of this study is to observe the change over time in a clinical finding between a patient's current and previous radiology reports. In this context, the problem is addressed as an NLP problem that enables clinical comparison between reports, rather than the individual classification of radiology reports.

### 3.1 Dataset

This study used the LUNGUAGE dataset published on PhysioNet (22, 23). This dataset is a structured and radiologist-annotated dataset developed for sequential chest radiograph interpretation tasks. This dataset was created from a subset of the MIMIC-CXR dataset (1, 24). The dataset is designed to be used for chest radiograph reports, not only at the single report level but also for sequential patient reviews. The dataset contains 1,473 chest radiograph reports from 230 patients. The data were labeled with over 17,000 expert-validated entities and 23,000 relationship-attribute pairs in 18 relationship types. The reports were created and validated by expert radiologists in the field.

The dataset includes radiology reports in both free-text and structured representations at the entity, relationship, and attribute levels. The official description of the dataset states that radiology reports contain detailed clinical observations and diagnostic reasoning that change over time, but existing evaluation metrics have limitations in capturing clinical semantics and temporal relationships. This demonstrates that the dataset is a unique resource for structured report generation and sequential report evaluation.

Each report in the dataset is decomposed into sentence-level clinical observations tagged with a section label. Three section types are present: Findings (find), Impression (impr), and History (hist). The models did not receive the raw full report; instead, for each observation the relevant structured fields were serialised into a compact observation text containing the section, sentence, entity and normalized entity, category, diagnostic status and certainty, location, morphology, distribution, measurement, severity, comparison, temporal attributes (new/stable/improved/worsened), study time, and sequence index. In this work, “entity” denotes a single annotated clinical concept as written in the report (e.g., “cardiac silhouette”); “normalized entity” is its canonical form (e.g., “cardiomegaly”); and the “target entity group” (equivalently “finding group”) is the set of observations of the same normalized entity for one patient across successive studies, which defines the longitudinal series whose transition is classified.

In this study, the dataset was used not for generating structured reports, but for detecting temporal novelty in consecutive reports. Each sample was considered to include current and previous study information for the same patient, the target clinical entity and finding group, and entity information between reports. The primary classification objective in the study is to determine which temporal state the target finding in the current report refers to in relation to the previous report. This structure, unlike single-report classification, relies on the model's interpretation of the clinical relationship between reports.

The dataset contains more than just the current report and current finding statement. It includes the time information from the previous report, the current time information, the target entity group, the entity from the previous report, and the entity from the current report. Because of this structure, the study is more complex than the classic text classification problem. The model is expected to determine whether a finding was not present in the previous report, and whether the statement in the report refers to the same clinical situation as the previous finding or a new situation. The appearance of a new finding in the current report that was not present in the previous report or that clinically differs from the previous finding is indicated by the “new” class label. This class is one of the most clinically important classes.

Temporal labels were derived automatically by comparing the previous and current annotated observations of the same entity group for a patient, following a fixed precedence. First, explicit annotation attributes were checked in order: a populated “worsened” attribute yielded WORSENED, “improved” yielded IMPROVED, “no change” yielded STABLE, and “onset” yielded NEW. If none applied, the free-text “comparison” attribute was scanned for direction keywords (e.g., increased/progressed/larger → WORSENED; decreased/smaller/less/resolved → IMPROVED; unchanged/stable/similar → STABLE; new/interval development/developed → NEW). If the label was still undetermined, the diagnostic-status transition was used: positive→negative was labelled RESOLVED, and negative→positive was labelled NEW. Pairs satisfying none of these rules were labelled UNKNOWN and excluded from the experimental pool. This precedence gives priority to explicit radiologist annotations over free-text cues and uses status transitions only as a fallback.

The suitability of the dataset for this study is based on three reasons. First, since the dataset consists of chest radiography reports, it is directly relevant to the clinical field of the study. Second, the dataset includes not only individual reports but also sequential reports and patient-level temporal relationships. Third, structured data at the entity and relationship level allows LLMs to assess not only their overall understanding of the report but also their temporal reasoning capacity in terms of findings. In these respects, the LUNGUAGE dataset is suitable for evaluating LLMs' capacity to interpret longitudinal radiology reports.

The dataset was used in a file-based format to ensure the reproducibility of experimental comparisons. The master data file and the entity dictionary were used as separate files. Operations such as data reading, sample creation, model input generation, output normalization, and results recording were structured under the project directory. With this structure, different models and prompt strategies were compared on the same sample. In this LLM-based evaluation, consistent data usage is crucial for reusability.

The data presented in Table 1 show the distribution used for the main experiment. After the data were excluded from the experiment, 1,500 samples were assigned to five basic temporal change classes. Because evaluating all seven models under five prompt strategies (35 configurations) over the full pool would require on the order of 3 × 10^5^ API calls, the comparative multi-model/multi-prompt evaluation reported in Tables 2–10 was carried out on a fixed, class-stratified random subset of 1,500 examples drawn from this pool, using a fixed random seed (42) for reproducibility. The subset preserves the ordering of the class distribution. The same 1,500 examples were presented to every model and every prompt strategy, so all configurations are compared on identical inputs. The highest percentage, 32.25%, belonged to the stable class with 484 samples. This class was followed by the new class with 415 samples (27.69%), the resolved class with 402 samples (26.78%), the worsened class with 108 samples (7.23%), and the improved class with 91 samples (6.05%).

**Table 1:** Class distribution of rule-based derived temporal instances.

Class	Sample Size	Ratio (%)
Stable	484	32.25
New	415	27.69
Resolved	402	26.78
Worsened	108	7.23
Improved	91	6.05
Total	1,500	100.00

**Table 2:** Configuration of prompt strategies.

Prompt Strategy	Original Prompt Structure
Zero-shot	You are evaluating temporal clinical reasoning in longitudinal chest x-ray reports. Compare the previous observation and the current observation. Return only valid JSON: {"label": “..”, “rationale”: “brief explanation”}. Allowed labels: NEW, IMPROVED, WORSENED, STABLE, RESOLVED, UNKNOWN (The UNKNOWN option was also presented as a dissenting label for situations with insufficient evidence in the study). Previous observation: {prev_observation} Current observation: {curr_observation}
Few-shot	You are evaluating temporal clinical reasoning in longitudinal chest x-ray reports. Compare the previous observation and the current observation. Return only valid JSON. Use these examples as guidance: Example 1: right pleural effusion is present → right pleural effusion is unchanged → STABLE. Example 2: no focal opacity → new left lower lobe opacity → NEW. Example 3: moderate pulmonary edema → mild pulmonary edema → IMPROVED. Example 4: small pleural effusion → large pleural effusion → WORSENED. Example 5: left pneumothorax → no pneumothorax → RESOLVED. Target case: Previous observation: {prev_observation} Current observation: {curr_observation}
Structured reasoning	Before deciding, internally check these clinical reasoning steps: 1. Was the current finding absent in the previous observation? 2. Was the previous finding present but now absent? 3. Is the same finding less severe? 4. Is the same finding worse? 5. Is the same finding unchanged? 6. Is there insufficient evidence? Do not exit the steps. Output only JSON. Previous observation: {prev_observation} Current observation: {curr_observation}
Memory-augmented	You are given a short clinical memory timeline before the current observation. Use the timeline to distinguish: a truly new finding, worsening of a known finding, stable persistence, improvement, resolution. Clinical memory timeline: {memory_context} Current observation: {curr_observation} Return only JSON.
Temporal graph	Represent the case as a temporal graph. Each node is an observation of the same entity group over time. The final node is the current observation. Compare the final node with the immediately preceding clinically relevant node. Temporal graph nodes: {graph_context} Return only JSON for the transition into the final node.

**Table 3:** Overall performance results of model-prompt combinations.

Model	Prompt	Accuracy	Macro-F1	Weighted-F1	New Class Recall
GPT-4.1	Few-shot	**0**.**8369**	**0**.**7888**	**0**.**8322**	**0**.**5385**
GPT-4.1	Zero-shot	0.8357	0.7874	0.8300	0.5200
GPT-4.1	Structured reasoning	0.8163	0.7729	0.8117	0.5000
GPT-4.1	Memory-augmented	0.8255	0.7826	0.8209	0.5000
GPT-4.1	Temporal graph	0.8082	0.7545	0.7977	0.4286
GPT-4.1-mini	Zero-shot	0.8125	0.7704	0.8071	0.5000
GPT-4.1-mini	Few-shot	0.8082	0.7670	0.8055	0.5000
GPT-4.1-mini	Structured reasoning	0.7823	0.7495	0.7836	0.5357
GPT-4.1-mini	Memory-augmented	0.8121	0.7719	0.8094	0.5185
GPT-4.1-mini	Temporal graph	0.8188	0.7756	0.8126	0.5000
GPT-4o	Zero-shot	0.8219	0.7685	0.8193	0.5000
GPT-4o	Few-shot	0.8231	0.7737	0.8198	0.5000
GPT-4o	Structured reasoning	0.7931	0.7486	0.7868	0.4643
GPT-4o	Memory-augmented	0.8188	0.7744	0.8158	0.5000
GPT-4o	Temporal graph	0.8188	0.7604	0.8106	0.4286
GPT-4o-mini	Zero-shot	0.7533	0.6983	0.7630	0.3214
GPT-4o-mini	Few-shot	0.7584	0.7012	0.7625	0.2857
GPT-4o-mini	Structured reasoning	0.7867	0.7515	0.7999	0.5000
GPT-4o-mini	Memory-augmented	0.7600	0.7236	0.7661	0.3571
GPT-4o-mini	Temporal graph	0.7133	0.6509	0.7149	0.3214
Gemini-2.5-flash	Zero-shot	0.8029	0.7679	0.8017	0.4615
Gemini-2.5-flash	Few-shot	0.7872	0.7410	0.7833	0.4815
Gemini-2.5-flash	Structured reasoning	0.7762	0.7281	0.7763	0.4643
Gemini-2.5-flash	Memory-augmented	0.7770	0.7291	0.7751	0.5000
Gemini-2.5-flash	Temporal graph	0.8099	0.7601	0.8083	0.5185
Claude Haiku 4.5	Zero-shot	0.7733	0.6990	0.7623	0.3929
Claude Haiku 4.5	Few-shot	0.7933	0.7286	0.7864	0.5000
Claude Haiku 4.5	Structured reasoning	0.7838	0.7089	0.7698	0.3462
Claude Haiku 4.5	Memory-augmented	0.7785	0.7097	0.7674	0.4074
Claude Haiku 4.5	Temporal graph	0.7600	0.6832	0.7434	0.3571
Claude Sonnet 4.6	Zero-shot	0.7586	0.6931	0.7452	0.3333
Claude Sonnet 4.6	Few-shot	0.7517	0.6860	0.7351	0.2917
Claude Sonnet 4.6	Structured reasoning	0.7586	0.6931	0.7452	0.3333
Claude Sonnet 4.6	Memory-augmented	0.7551	0.6832	0.7429	0.3200
Claude Sonnet 4.6	Temporal graph	0.7619	0.6807	0.7476	0.3333

Bold values highlight the key performance results emphasized in the corresponding Table.

**Table 4:** Average performance values according to prompt strategies.

Prompt Strategy	Average Accuracy	Average Macro-F1	Average Weighted-F1	Average New Recall
Few-shot	**0**.**7941**	**0**.**7409**	0.7893	**0**.**4425**
Zero-shot	0.7940	0.7407	**0**.**7898**	0.4327
Structured reasoning	0.7853	0.7361	0.7819	0.4491
Memory-augmented	0.7896	0.7392	0.7854	0.4433
Temporal graph	0.7844	0.7236	0.7764	0.4125

Bold values highlight the key performance results emphasized in the corresponding Table.

**Table 5:** Comparison of new class precision, recall, F1, and macro-F1 models.

Order	Model	Prompt	New Precision	New Recall	New F1	Macro-F1
1	GPT-4.1	Few-shot	**1**.**0000**	**0**.**5385**	**0**.**7000**	**0**.**7888**
2	GPT-4.1-mini	Structured reasoning	0.7895	0.5357	0.6383	0.7495
3	GPT-4.1	Zero-shot	0.9286	0.5200	0.6667	0.7874
4	Gemini-2.5-flash	Temporal graph	0.8750	0.5185	0.6512	0.7601
5	GPT-4.1-mini	Memory-augmented	0.8235	0.5185	0.6364	0.7719
6	GPT-4o	Few-shot	1.0000	0.5000	0.6667	0.7737
7	GPT-4o	Zero-shot	1.0000	0.5000	0.6667	0.7685
8	GPT-4o-mini	Structured reasoning	1.0000	0.5000	0.6667	0.7515
9	Claude Haiku 4.5	Few-shot	1.0000	0.5000	0.6667	0.7286
10	Gemini-2.5-flash	Memory-augmented	0.9333	0.5000	0.6512	0.7291

Bold values highlight the key performance results emphasized in the corresponding Table.

**Table 6:** Class-based precision, recall, and F1 values for the best model-prompt combination (GPT-4.1 + few-shot).

Class	Precision	Recall	F1-score
New	**1**.**0000**	0.5385	0.7000
Improved	0.6377	0.9670	0.7686
Worsened	0.5027	0.8519	0.6323
Stable	0.8769	**0**.**9711**	0.9216
Resolved	0.9095	0.9502	**0**.**9294**

Bold values highlight the key performance results emphasized in the corresponding Table.

**Table 7:** Sample data for four types of errors.

Error Type	Target Finding Group	Previous Observation	Current Observation	Real Label	Model Prediction	Model Rationale
New Finding Missed	Cardiac silhouette enlarged top-normal to mildly	Section: find sentence: the heart is top-normal in size. entity: heart normalized_entity: heart category: pf diagnostic_status: negative diagnostic_certainty: definitive measurement: top-normal in size study_time: 2189.11.10 14:13 sequence: 6	Section: find sentence: the cardiac silhouette is top-normal to mildly enlarged. entity: cardiac silhouette normalized_entity: cardiac silhouette category: pf diagnostic_status: positive diagnostic_certainty: definitive measurement: enlarged severity: top-normal to mildly study_time: 2189.11.11 21:40 sequence: 7	New	Worsened	The heart size has progressed from top-normal to mildly enlarged, indicating an increase in size.
Directionality Error	Opacity right lung diffusely increased	Section: impr sentence: diffusely increased opacity throughout the right lung is related to a chronic loculated right effusion and multifocal opacities worrisome for pneumonia. entity: opacity normalized_entity: opacification category: pf diagnostic_status: positive diagnostic_certainty: definitive location: right lung distribution: diffusely worsened: increased study_time: 2189.11.10 14:13 sequence: 6	Section: find sentence: there is diffusely increased opacity throughout the right lung, which is in part related to a chronic loculated effusion. entity: opacity normalized_entity: opacification category: pf diagnostic_status: positive diagnostic_certainty: definitive location: right lung distribution: diffusely worsened: increased study_time: 2189.11.10 14:13 sequence: 6	Worsened	Stable	The diffusely increased opacity throughout the right lung persists with similar description and extent; no interval change is indicated.
Resolution Missed	Heart size upper limits of normal	Section: find sentence: cardiomegaly is stable. entity: cardiomegaly normalized_entity: cardiomegaly category: pf diagnostic_status: positive diagnostic_certainty: definitive no_change: stable study_time: 2174.6.4 15:45 sequence: 2	Section: find sentence: the heart size is at the upper limits of normal. entity: heart size normalized_entity: heart size category: pf diagnostic_status: negative diagnostic_certainty: definitive measurement: upper limits of normal study_time: 2174.6.10 10:02 sequence: 3	Resolved	Improved	Previously, cardiomegaly (enlarged heart) was present and stable; Currently, the heart size is described as at the upper limits of normal, indicating improvement from abnormal to borderline/normal.
Other Temporal Error	Pleural effusion left massively increased pre-existing	Section: impr sentence: small bilateral pleural effusion, left greater than right, unchanged over nine hours. entity: pleural effusion normalized_entity: pleural effusion category: pf diagnostic_status: positive diagnostic_certainty: definitive location: bilateral measurement: small comparison: left greater than right no_change: unchanged study_time: 2151.3.8 23:16 sequence: 10	Section: impr sentence: lung bases show persistent small-to-moderate bilateral pleural effusion. entity: pleural effusion normalized_entity: pleural effusion category: pf diagnostic_status: positive diagnostic_certainty: definitive location: bilateral severity: small-to-moderate no_change: persistent study_time: 2151.3.8 23:53 sequence: 11	Stable	Worsened	The severity of bilateral pleural effusion increased from small to small-to-moderate.

**Table 8:** Distribution of model-prompt combinations according to error types.

Model	Prompt	New Finding Missed	Directionality Error	Resolution Missed	Other Temporal Error	Total Error
GPT-4.1	Zero-shot	12	8	1	2	23
GPT-4.1	Few-shot	12	8	2	1	23
GPT-4.1	Structured reasoning	14	9	1	3	27
GPT-4.1	Memory-augmented	14	8	1	3	26
GPT-4.1	Temporal graph	16	5	4	3	28
GPT-4.1-mini	Zero-shot	14	8	2	3	27
GPT-4.1-mini	Few-shot	14	9	2	3	28
GPT-4.1-mini	Structured reasoning	13	11	3	5	32
GPT-4.1-mini	Memory-augmented	13	8	3	4	28
GPT-4.1-mini	Temporal graph	14	7	2	4	27
GPT-4o	Zero-shot	14	9	2	1	26
GPT-4o	Few-shot	14	9	2	1	26
GPT-4o	Structured reasoning	15	11	1	3	30
GPT-4o	Memory-augmented	14	7	2	4	27
GPT-4o	Temporal graph	16	6	4	1	27
GPT-4o-mini	Zero-shot	19	15	1	2	37
GPT-4o-mini	Few-shot	20	12	2	2	36
GPT-4o-mini	Structured reasoning	14	16	1	1	32
GPT-4o-mini	Memory-augmented	18	14	1	3	36
GPT-4o-mini	Temporal graph	19	16	4	4	43
Gemini-2.5-flash	Zero-shot	14	11	1	1	27
Gemini-2.5-flash	Few-shot	14	11	4	1	30
Gemini-2.5-flash	Structured reasoning	15	12	4	1	32
Gemini-2.5-flash	Memory-augmented	14	13	4	2	33
Gemini-2.5-flash	Temporal graph	13	8	3	3	27
Claude Haiku 4.5	Zero-shot	17	10	6	1	34
Claude Haiku 4.5	Few-shot	14	9	7	1	31
Claude Haiku 4.5	Structured reasoning	17	9	5	1	32
Claude Haiku 4.5	Memory-augmented	16	10	6	1	33
Claude Haiku 4.5	Temporal graph	18	9	8	1	36
Claude Sonnet 4.6	Zero-shot	16	14	4	1	35
Claude Sonnet 4.6	Few-shot	17	14	4	1	36
Claude Sonnet 4.6	Structured reasoning	16	14	4	1	35
Claude Sonnet 4.6	Memory-augmented	17	11	5	3	36
Claude Sonnet 4.6	Temporal graph	18	9	7	1	35

**Table 9:** Bootstrap confidence intervals for the most successful model-prompt combinations.

Model	Prompt	Macro-F1 Mean	Macro-F1 95% CI	New Recall Mean	New Recall 95% CI
GPT-4.1	Zero-shot	0.7836	0.6996-0.8586	0.5152	0.3156-0.7083
GPT-4.1	Few-shot	0.7827	0.6925-0.8606	0.5384	0.3500-0.7242
GPT-4.1	Memory-augmented	0.7771	0.6996-0.8500	0.4993	0.3200-0.6765
GPT-4.1-mini	Temporal graph	0.7722	0.6952-0.8515	0.5043	0.3180-0.6800
GPT-4o	Memory-augmented	0.7711	0.6868-0.8497	0.4934	0.2916-0.6858
GPT-4o	Few-shot	0.7665	0.6794-0.8443	0.5039	0.3043-0.6924
GPT-4.1	Structured reasoning	0.7658	0.6828-0.8411	0.5018	0.3158-0.6818
GPT-4.1-mini	Memory-augmented	0.7672	0.6803-0.8473	0.5143	0.3214-0.7038
GPT-4.1-mini	Zero-shot	0.7639	0.6731-0.8428	0.5014	0.3077-0.6842
GPT-4o	Zero-shot	0.7651	0.6851-0.8405	0.4987	0.3200-0.6897

**Table 10:** Paired mcNemar comparisons among the leading configurations.

Comparison (GPT-4.1 few-shot vs.)	n pairs	b	c	*p*
GPT-4.1 + zero-shot	1360	0	1	1.00
GPT-4.1 + memory-augmented	1410	3	4	1.00
GPT-4.1-mini + temporal graph	1400	6	6	1.00
GPT-4o + memory-augmented	1400	5	6	1.00
GPT-4o + few-shot	1400	2	1	1.00

To make the temporal novelty detection task concrete, Table 11 presents a single de-identified example drawn from the dataset. Each instance pairs the previous and current observations of the same target entity group for one patient, together with their study times, and is assigned one of the five temporal labels by the rule-based procedure. In this example, the target finding (pneumonia) is absent in the earlier study but reported as a new development in the later study of the same patient; the diagnostic status therefore shifts from negative to positive, and the label NEW is assigned. This illustrates how each example encodes not merely a single report but the clinical relationship between two consecutive reports, which is what the models are subsequently asked to interpret.

**Table 11:** A de-identified example instance from the dataset.

**Field**	**Value**
Target entity group	Pneumonia (ventilated parts of the lung parenchyma)
Previous study (time)	s58992648 · 2151-02-10 06:02 - Findings: lungs clear; no focal consolidation; pneumonia absent (diagnostic_status: negative)
Current study (time)	s51235553 · 2151-02-22 08:23 - Findings: possible new pneumonia in ventilated parts of the lung parenchyma (diagnostic_status: positive)
Entity/normalized	Pneumonia/pneumonia
Temporal information	Negative → positive across two consecutive studies of the same patient
Gold temporal label	NEW

### 3.2 Models

The selection of models for this study was not based solely on the highest capacity criterion. Instead, a model set was created that included different model families, large and small model variants, long context support, performance and cost balance, and prompt tracking capacity. Seven models from the OpenAI, Anthropic Claude, and Google Gemini providers were included in the study. These models were selected as GPT-4.1, GPT-4.1-mini, GPT-4o, GPT-4o-mini, Claude Sonnet 4.6, Claude Haiku 4.5, and Gemini 2.5 Flash. These models are suitable for comparing clinical text comparison and temporal reasoning tasks for LLMs in terms of model scale and intended use.

The rationale for the model set is stated once here and not repeated per model: the set spans three providers (to avoid single-ecosystem conclusions), pairs high-capacity models with smaller/faster variants (to test whether a lighter model suffices), and balances cost, latency, and scalability against peak accuracy. All models support long context and structured, instruction-constrained output, which the task requires. Only the distinguishing characteristics of each model are given below.

All models used in this study are closed-source commercial LLMs. This means that details such as the number of layers in the models, total parameters, scope of the training dataset, data content, data cleaning, and post-training alignment are not publicly shared.

In this study, GPT-4.1, GPT-4.1-mini, GPT-4o, GPT-4o-mini, Claude Sonnet 4.6, Claude Haiku 4.5, and Gemini 2.5 Flash models were used. The GPT-4.1 model is one of the high-capacity models in the GPT model family offered by OpenAI. OpenAI uses the expression “smartest non-reasoning model” for this model. It is stated that this model is strong in instruction following, tool use, and tasks involving extensive knowledge in different fields. The model can take text and images as input. The generated text output has a context window at the level of 1 million tokens. Compared to other models in the GPT family, it has shown improvement, especially in coding, instruction following, and long context understanding tasks (25–27).

The GPT-4.1-mini model is a smaller, faster, and lower-cost member of the GPT-4.1 model. Like the GPT-4.1 model, the GPT-4.1-mini model accepts text and image inputs. The model is powerful in generating text output, instruction following, and tool use. This model was developed for applications requiring lower latency and cost (28).

The GPT-4o model is one of the multimodal and high-capacity models in the OpenAI model family. This model accepts text and image inputs. The GPT-4o model is a powerful model that produces text as output, supports structured outputs, and is suitable for many tasks (29). In this study, the multimodal and general-purpose powerful model structure of the GPT-4o model has made it an important comparative model in NLP studies (30, 31).

The GPT-4o-mini model is a model developed for rapid and low-cost use within the GPT-4o model family. This model accepts text and image input, produces text output, and supports structured outputs. The model has a 128,000-token context window and a maximum token output of 16,384 (29). With this model, outputs obtained from larger models are distilled to produce similar results at a lower cost and with less delay.

Claude Sonnet 4.6 is one of the high-capacity models in the Anthropic Claude model family. This model is introduced as an improved model in the fields of coding, long-context reasoning, agent planning, and information design (32). This model has a context window of 1 million tokens.

The Claude Haiku 4.5 model is a small-sized model created by Anthropic for low-cost and fast use. This model offers a lower pricing strategy compared to the Sonnet model family. The model can be used as a cost-sensitive and scalable model requiring a fast response (33).

The Gemini 2.5 Flash model is a model created by Google's Gemini provider, offering a balanced price-performance ratio. The model accepts text, image, video, and audio input and produces text data. It has an input limit of 1,048,576 tokens and a maximum output limit of 65,535 tokens (34).

## 4 Experimental setup

Experimental studies were conducted in the Google Colab environment. The study utilized an NVIDIA A100 GPU as the hardware accelerator. GPU acceleration was effective in data preparation, output processing, metric calculation, bootstrap sampling, and graphical analysis processes. Data files and results used during the study were stored on Google Drive. This ensured the preservation of intermediate outputs during the experiments.

Table 12 presents the parameters and settings for the models used in the study. All models were called via API. The models used in the study are closed-source API models. The models were not trained locally, and no fine-tuning process was applied. Five labels (new, improved, worsened, stable, resolved) constitute the clinical outcome domain in the study. The UNKNOWN option was also presented as a dissenting label for situations with insufficient evidence in the study; UNKNOWN responses were excluded before metric calculation. This information could not be considered as a sixth clinical category.

**Table 12:** Model parameters.

Parameter	Value Used	Explanation
Work environment	Google Colab	The cloud-based environment in which the experiments were conducted
Hardware accelerator	NVIDIA A100 GPU	GPUs are used in data processing, analysis, and visualization processes.
Data storage	Google Drive	The environment where data files and experimental outputs are stored.
Model access	API	The models were not run locally, but called via provider APIs.
Training/fine-tuning	None	No fine-tuning has been done on the models.
Type of inference	Prompt-based inference	The models were evaluated using different prompt strategies.
Heat	0	Deterministic and consistent output production.
Maximum output length	800 tokens	Responses should remain short, separable, and task-oriented.
Output format	Structured JSON	Ticket
Tag set	New, Improved, Worsened, Stable, Resolved	Temporal state classes
Operation strategy	Sustainable structure	Completed calls should not be re-operated.
Recording format	Row-based recording	Storing each model response at the sample level.

The experimental study process involved comparing the same data samples using different models and prompt strategies. Each sample in the dataset included the target clinical entity group, time information from the current and previous studies, and a temporal label from current and previous chest radiography reports of the same patient. The expected task of the model in this study was to determine the temporal status of the target finding in the context of the given current and previous reports. This was done to interpret the clinical and temporal relationships between the two reports, unlike the single-text classification of the study.

Two distinct labelling processes are used and should not be conflated. The gold labels were produced by the deterministic rule-based procedure. At inference, the model was given the closed label set and, depending on the prompt strategy, either the labels alone (zero-shot) or the labels together with worked examples or step-by-step decision cues (few-shot, structured reasoning); it determined the label through its own reasoning over the two observations, and the prompt did not supply the gold rule that generated the reference label. The task therefore evaluates the model's temporal reasoning against an independently derived reference, not its ability to replay a rule it was given.

In this study, all models were run under the same experimental settings. The goal was to generate a structured and decomposable output from the models. This output allows for a common evaluation of the outputs of models from different providers. The output format of the models was designed to include the model's class decision. This structure enables both automatic evaluation of model responses and examination of error analysis.

The outputs that the models will produce are limited to the labels new, improved, worsened, stable, or resolved. These labels indicate the key temporal states to be examined between successive reports. The “new” class indicates a new finding that was not present in the previous report or that clinically differs from the situation described in the previous report. The “improved” class indicates that the finding in a report has decreased or improved compared to the previous report. The “worsened” class indicates that the finding has increased or worsened compared to previous reports. The “stable” class indicates that there has been no significant change in the finding compared to previous reports. The “resolved” class indicates that a finding present in previous reports has disappeared in the current report.

In model calls, the temperature parameter is set to 0 for all models to ensure deterministic output generation. In LLM, the temperature parameter controls the randomness in the model's output distribution. High values for this parameter can produce more varied but misleading responses. Low values yield more consistent and repeatable outputs. In clinical classification and labeling studies, the goal is not to generate creative responses but to generate consistent decisions. For this reason, the temperature parameter is set to 0. To prevent model responses from becoming unnecessarily lengthy explanations, the maximum output length is limited to 800 tokens.

Table 13 presents the categories and experimental roles of the models, indicating the rationale for their inclusion in the study. This data demonstrates that the study did not rely on a single provider or scale of models. Models were categorized as high-capacity, mini or fast models, long-context, and cost-performance balanced. This structure allows the study to evaluate models not only with the strongest model but also those representing different use cases.

**Table 13:** Model-based API configuration.

Model	Provider	Model Category	Experimental Role
GPT-4.1	OpenAI	High-capacity general model	Powerful OpenAI model
GPT-4.1-mini	OpenAI	Efficient/mini model	Cost-performance comparison
GPT-4o	OpenAI	Multi-mode powerful model	GPT-4o family comparison
GPT-4o-mini	OpenAI	Fast and low-cost model	Comparison of efficient models
Claude Sonnet 4.6	Anthropic	High-capacity Claude model	Comparison between providers
Claude Haiku 4.5	Anthropic	The fast and cost-effective Claude model.	Cost-sensitive comparison
Gemini 2.5 Flash	Google	Long-term and rapid models	Google model provider comparison

Each of the five prompt strategies (Table 14) was applied independently. For every combination of model, prompt strategy, and example, a single self-contained prompt was sent to the model in one turn, and one prediction was obtained; there was no multi-turn exchange, and no prompt depended on the output of another. Each model, strategy, and example run was recorded under a unique run identifier and scored independently, so the 35 model–prompt configurations are fully crossed and mutually independent. In the zero-shot strategy, the model was given a task description, labels, and decision rules. In the few-shot strategy, the model was presented with limited sample decisions, and the aim was to make the label boundaries clearer. In the structured reasoning strategy, the model was asked to evaluate the relationship between the current report and the previous report in specific steps. In the memory-augmented strategy, the context of previous reports was presented as a more distinct clinical memory. In the temporal graph strategy, current and previous observations related to the same target entity were structured as temporal graph nodes. The use of five different prompt strategies shows that the study was not based solely on model comparison but also on the evaluation of prompts. This structure is based on the assumption that in the temporal novelty detection task of LLMs, different results can be obtained not only with the capacity of the model but also with the context and the structure of the decision instructions. This provides a more comprehensive evaluation of the study that takes into account both model selection and prompt design.

**Table 14:** Prompt strategies and their purposes.

Prompt Strategy	Basic Structure	Experimental Purpose
Zero-shot	Job description and tag set	To measure the model's ability to understand tasks without examples.
Few-shot	Job description + sample decisions	Defining label boundaries with examples.
Structured reasoning	Step-by-step clinical monitoring guidelines	Structuring temporal reasoning
Memory-augmented	Presenting the prior context as clinical memory.	Evaluating the impact of prior knowledge on the decision.
Temporal graph	Presenting previous and current observations with temporal nodes.	Clearly represent the temporal relationship between reports.

Table 2 presents the model input for prompt methods. For all methods, only JSON output was requested from the model. The classification space was limited to the labels new, improved, worsened, stable, and resolved. In the prompt templates, UNKNOWN was only included as an abstention option in cases of insufficient evidence. This was structured in a way that would not create a sixth clinical class. It was removed before such an assessment. In the few-shot method, sample decisions for each class were presented to the model. In the structured reasoning method the decision-making process involved clinical comparisons that the model was expected to follow internally. In the memory-augmented and temporal graph methods, contextual evaluation was aimed at using patient history and temporal node structure.

To enable automatic evaluation of the outputs generated by the model, responses were requested in a structured format. Each model was required to explicitly specify the class label when generating its output. Different variations in labels may occur in the model outputs. To prevent this, output normalization was applied. For example, data containing expressions such as “unchanged”, “no change”, and “same” were assigned to the stable class; expressions such as “worse”, “progressed”, and “increased” were assigned to the worsened class; and expressions such as “resolved”, “no longer present”, and “disappeared” were assigned to the resolved class.

The study employed a fault-tolerant and recurring approach. A unique experiment ID was generated for each model-prompt-sample combination. A structure was created to prevent completed experiments from being rerun. This method ensures that experiments resume from where they left off in case of quota limits, service errors, connection interruptions, or timeouts that may be encountered in API-based experiments. This protects completed results and prevents incomplete or erroneous calls from being rerun.

Errors that may occur in API calls are logged in the project directory. Situations such as service errors, quota exceeding, blank responses, unparsable data, and timeouts on the API are monitored in addition to model predictions. This prevents the model's actual classification performance from being confused with API-related technical errors. Performance analyses of the study results are based on examples where the model produced output and the label could be separated.

**Figure 1:**
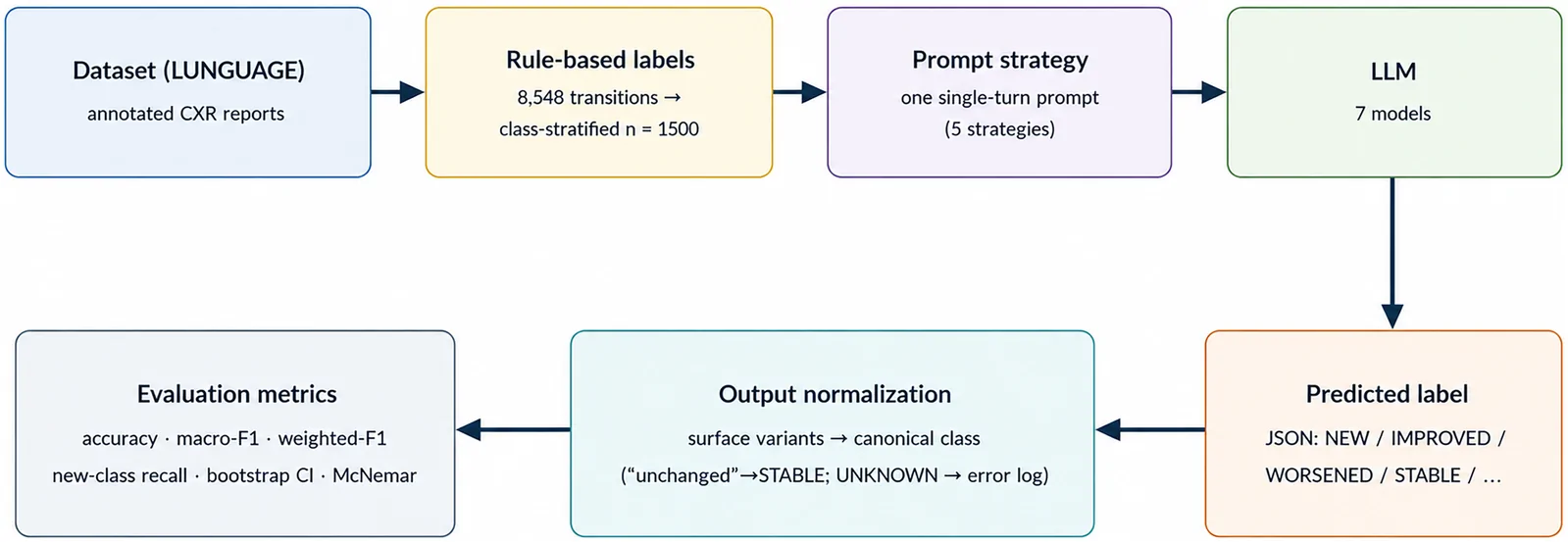
Experimental pipeline.

In the experimental study, result generation and result analysis were conducted separately. In the first stage of the study, predictions were obtained from the models and recorded as samples in the project directory. In the second stage, the model predictions were normalized and made comparable to standard labels. In the third stage, metrics such as accuracy, macro-F1, weighted-F1, class-based precision, recall, F1-score, and new class recall were calculated. The experimental pipeline is presented in Figure 1.

## 5 Findings

The findings obtained from the experimental study were evaluated using overall model-prompt performance, the effect of prompt strategies, new class recall, distribution of error types, class-based confusion patterns, and bootstrap confidence intervals. The aim of this method is not only to identify the model with the highest success rate but also to reveal the strengths and weaknesses of the models in temporal clinical reasoning.

The overall performance of all model-prompt combinations evaluated in the study is presented in Table 3. These results show that the models differ not only in terms of provider or model family, but also in terms of prompt strategies. Accuracy was computed as the number of examples whose predicted label equals the gold label, divided by the number of valid (non-UNKNOWN) predictions for that configuration; it is a micro-level rate dominated by the most frequent classes. The macro-F1 metric gives equal weight to each class, making the model's success in underrepresented classes more visible. The weighted-F1 metric takes into account the class distribution. This shows the performance in the overall data structure. The new class recall metric shows the sensitivity in detecting new findings, which is clinically important information. When the results are examined, it is observed that the most successful model-prompt combinations are GPT-4.1 and the few-shot method. This result shows that the task is sensitive not only to the model's capacity but also to the model's prompt structure. While some models showed successful results for the weighted-F1 metric, they were more limited in the macro-F1 metric. This difference indicates that the model showed successful results in dominant classes, but was limited in less represented or clinically more difficult classes. Therefore, in tasks with unbalanced class distribution, the macro-F1 metric can be more discriminatory than the accuracy value.

A model with a high weighted-F1 value can achieve a high score by correctly predicting frequently occurring labels, but the macro-F1 metric highlights failures in rare classes; model performance should therefore be evaluated using multiple metrics, not a single one. Rather than a separate ranking table (which merely reorders Table 3), the three best configurations by macro-F1 are noted inline: GPT-4.1 with few-shot (0.7888), zero-shot (0.7874), and memory-augmented (0.7826) prompts.

The presence of different prompt strategies of the same model near the top indicates that model capacity is a determining factor, while the presence of different models with the same prompt strategy indicates that prompt structure also matters. Clinically, a model with a high macro-F1 score but a low new class recall value carries the risk of missing newly emerging findings; the most successful model should be evaluated not only by the overall score but also by the clinical sensitivity metric.

Table 4 presents the average effect of prompt strategies on model performance. The results here show which prompt structure is more effective on the task, regardless of the model family. The average performance values of the prompt strategies show that the results obtained are similar. The highest value in terms of average accuracy was obtained with the memory-augmented method. This result shows that presenting the previous context more clearly to the model can increase the accuracy rate. On the macro-F1 metric, the highest values are seen in the zero-shot and few-shot methods. This indicates that simpler prompt structures are competitive in maintaining balance between classes. On the new class recall metric, the highest performance was obtained with the structured reasoning method. This result shows that structured and step-by-step guidance of the model is effective in identifying new findings. The temporal graph method generally has the lowest values. This indicates that presenting the temporal relationship graphically or with a more complex presentation does not improve performance and, in some models, complicates the decision-making process.

Table 5 presents the precision, recall, new F1, and macro-F1 values at the model-prompt level for the new class label, one of the most clinically significant classes. In this study, recall in the new class was specifically addressed because it indicates the model's ability to detect new findings. Missing a newly emerging finding such as pneumonia, effusion, or consolidation in a clinical trial can pose a significant risk for follow-up. Therefore, this metric was considered an important indicator in evaluating the clinical sensitivity of the model. In the new class F1 metric, the most successful model-prompt combination was GPT-4.1 and few-shot. This combination achieved a precision of 1.0000, a recall of 0.5385, and a new F1 value of 0.7000. These results demonstrate that the most balanced outcome is obtained in detecting new findings without generating false positives. The second-ranked combination is GPT-4.1-mini and structured reasoning. This result shows that a smaller model achieves stronger performance with structured reasoning. The GPT-4.1 and zero-shot combination is significant in terms of its success in macro-F1 and precision values. Examining the data in the table, it is seen that the precision values are generally high for the new class, but the recall values are more limited. This indicates that the models mostly make correct decisions in predictions, but some new findings may be missed. This is clinically important because missing a newly developed radiological finding can pose a risk to patient follow-up.

The best model-prompt combination in overall success was GPT-4.1 and few-shot. Class-based performance for this combination is presented in Table 6. The results show that the model performed strongly, particularly in the resolved, stable, and improved classes. In the resolved class, high precision, recall, and F1-score values indicate that the model can distinguish resolved findings. In the stable class, the high recall value shows that the model successfully detects unchanged findings. In the improved class, the high recall value proves that findings indicating improvement were successfully recognized. In the new class, the precision value is very high. However, the in-class recall value is low. This result indicates that the model made correct predictions for the new class but missed some new findings. In the worsened class, recall is relatively high, but the precision value is limited. This indicates that some false positives occurred regarding the worsening of the findings.

**Figure 2:**
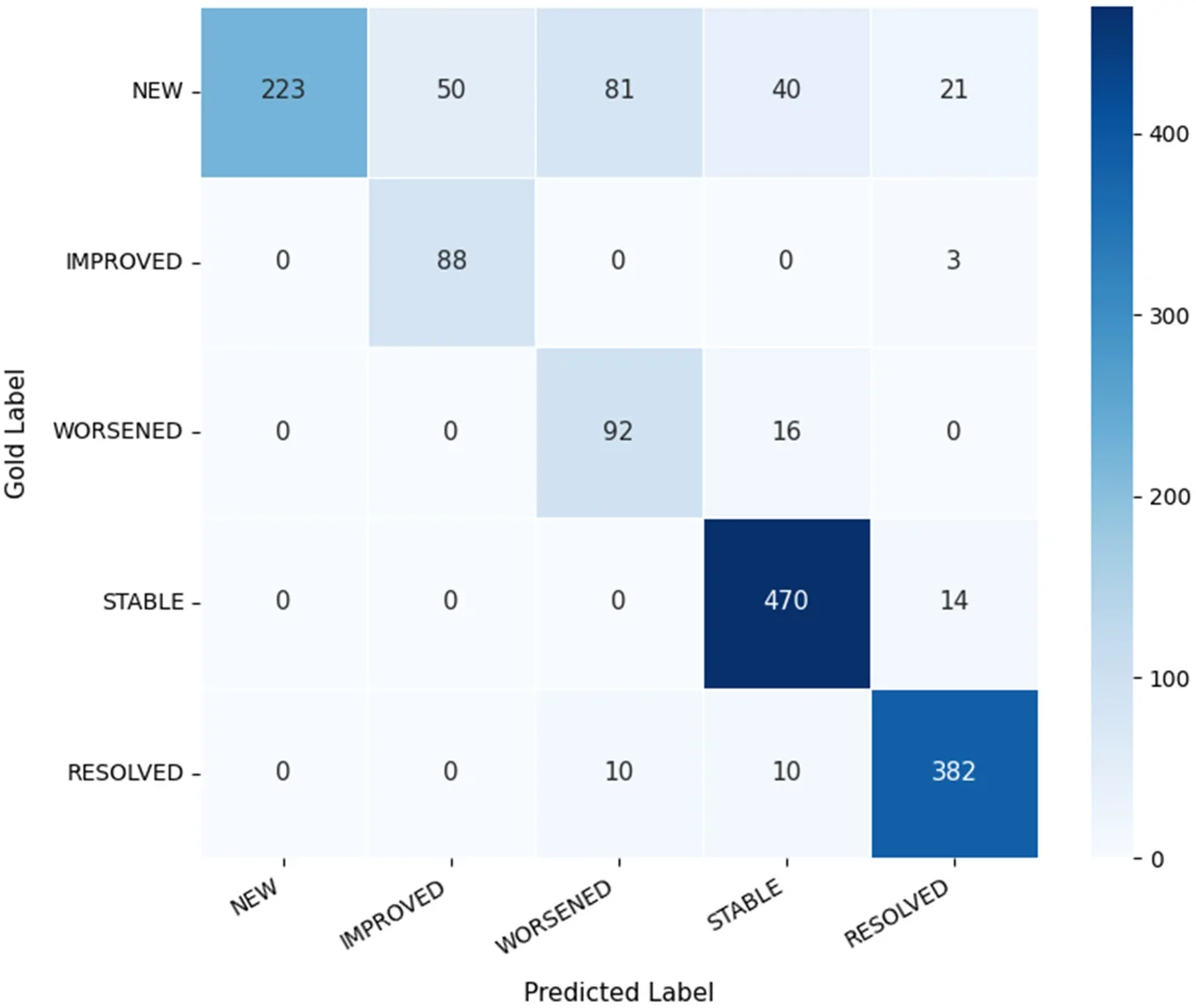
Confusion matrix for GPT-4.1 + few-shot configuration.

Figure 2 presents the class-based distribution of the GPT-4.1 and few-shot configuration. The confusion matrix generally produced the highest correct classification density in the stable and resolved classes. 470 correct predictions were observed in the stable class, 382 in the resolved class, and 223 in the new class. Some of the samples in the new class were classified as stable. This indicates that the model struggles to distinguish between newly emerging findings and those that remain unchanged. A similar situation is observed in the prediction of 88 data points in the resolved class as improved. This suggests that findings that have completely disappeared and those showing partial improvement share semantic similarities.

Table 7 presents the clinical context equivalents of model errors. For the new finding missed error type, the model interpreted the change from a negative to a positive finding as worsened instead of new class. For the directionality error type, despite the explicit indication of increased in the worsened domain, the model labeled the change as stable. In the resolution missed error type, the change from a positive cardiomegaly finding to a negative or normal range was evaluated as improved instead of resolved. In the other temporal error type, despite the indications of no change and persistent, the model classified the small difference in severity as worsened.

**Figure 3:**
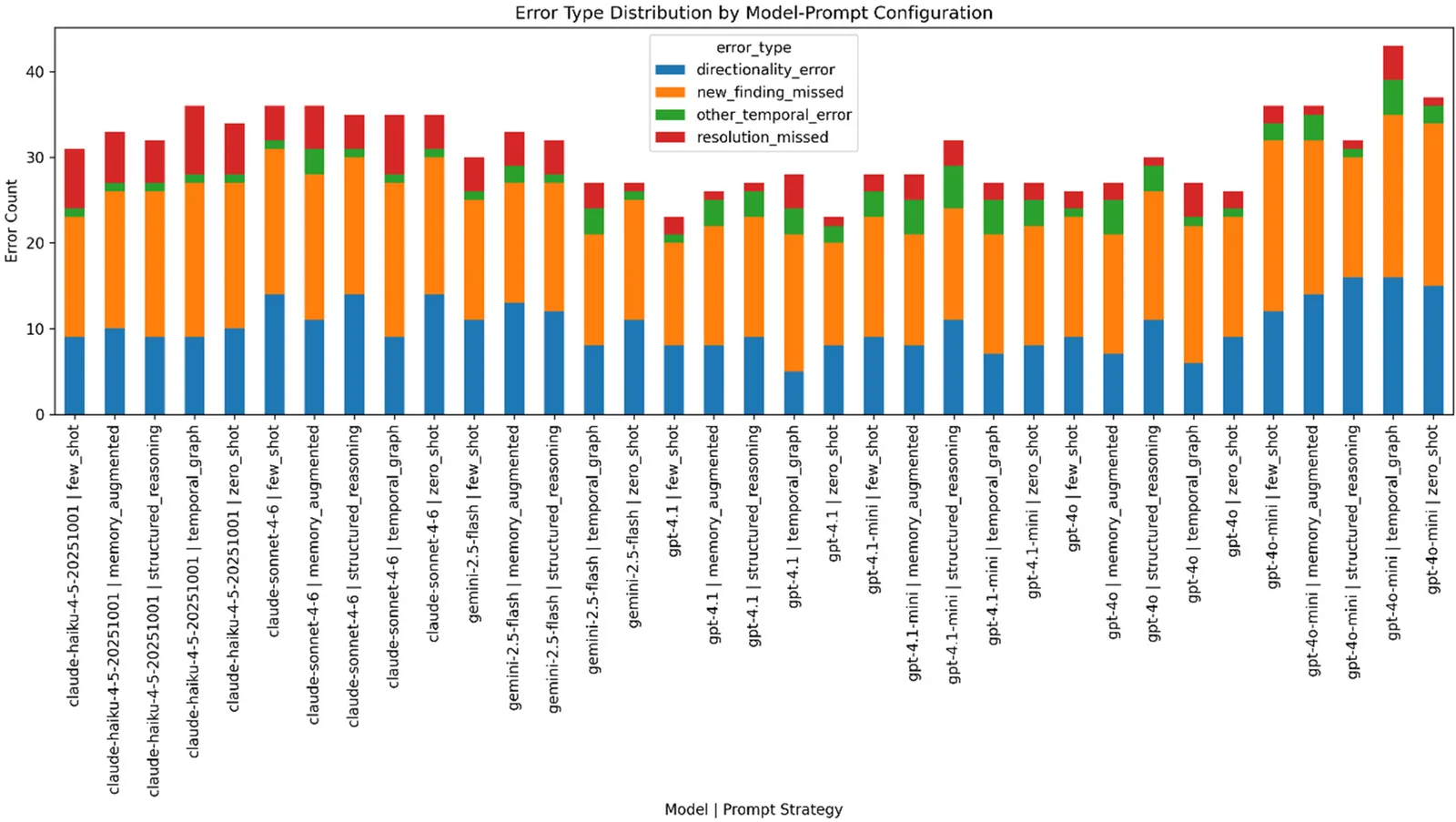
Stacked graph of error types according to model-prompt combinations.

Table 8 and Figure 3 present the distribution of model-prompt combinations according to error types. Errors were evaluated in four main groups. The new finding missed error type occurs when the model fails to detect a new finding that was not present in the previous report but appears in the current report. Directionality error refers to situations where the model misinterprets the direction of temporal change. An example of this error type is evaluating an improving finding as worsening or stable. Resolution missed indicates the failure to recognize a finding that was present in the previous report but not in the current report as resolved. Other temporal error represents situations that do not directly fall into the three error types mentioned above, but involve misinterpreting the temporal relationship between reports. Table 8 and Figure 3 show that the most dominant error type is new finding missed. In combinations, this error type ranges from 12 to 20. The lowest error value (12) is observed in the GPT-4.1—zero-shot and GPT-4.1—few-shot combinations. The highest error value was obtained with the GPT-4o-mini-few-shot combination, at 20. This data indicates that the models tend to miss newly emerging findings. The Directionality Error ranges from 5 to 16. The lowest value of 5 is seen in the GPT-4.1-temporal graph combination. The highest error value of 16 was observed in the GPT-4o-mini-structured reasoning and GPT-4o-temporal graph combinations. This shows that some small model combinations have difficulty determining the direction of change. The Resolution Missed error is more limited. This type of error ranges from 1 to 7. The highest error value of 7 was observed in the Claude Haiku 4.5—few-shot and Claude Sonnet 4.6—temporal graph combinations. This data shows that the missing of resolved findings is less common than the missing of new findings. The other temporal error type is generally more limited compared to other error types. This error type takes values between 1 and 5. The highest error of 5 was observed in the GPT-4.1-mini—structured reasoning combination. This indicates that errors generally occur in the form of missing new findings or misinterpreting the direction of change. The best results in terms of total error direction were obtained with the GPT-4.1—zero-shot and GPT-4.1—few-shot combinations. It is observed that errors are generally concentrated on new finding missed and directionality error. This shows that increasing the sensitivity to new findings and the direction of change is necessary in model improvements.

Table 9 presents the bootstrap confidence intervals for the best-performing model-prompt combinations. In LLM studies, models are generally ranked based on a single point estimate. The sensitivity of point estimates is particularly important in tasks where the class distribution is unbalanced or the sample size is limited. Bootstrap confidence intervals are important in determining the level of uncertainty in model performance. Table 9 shows the width of the confidence intervals and the uncertainty in predicting the performance of model-prompt combinations. The closeness of the macro-F1 confidence intervals of two models does not only indicate the superiority of one model over the point estimate. If the confidence intervals are divergent, the difference in model performance can be interpreted more strongly. The highest mean value in terms of bootstrap macro-F1 is 0.7836 in the GPT-4.1—zero-shot combination. In this combination, the macro-F1 confidence interval was calculated as 0.6996–0.8586. The second highest value is in the GPT-4.1—few-shot combination, which reached a macro-F1 value of 0.7827. This result demonstrates that the GPT-4.1 model produces stable and robust results for incoming classification using both few-shot and zero-shot methods.

A McNemar test was applied to the bootstrap confidence interval. Table 10 presents the results of comparing the best configuration in the study, GPT-4.1 + few-shot, with the next five successful configurations. The testing process was performed with sample-level matched accuracy. Samples in which both configurations produced a valid label were included in the comparison. Out-of-conformity estimates were low in number and balanced (b, c ≤ 6). No statistically significant difference was observed in the comparisons (*p* ≈ 1.0). The obtained results are consistent with the Macro-F1 confidence intervals presented in Table 9. The data from the two tables showed that the most successful GPT-4.1 configuration formed a statistically indistinguishable upper group rather than a single configuration showing absolute superiority. The difference between the best model and the weakest configuration (e.g., GPT-4o-mini + temporal graph) produced a significant result.

**Figure 4:**
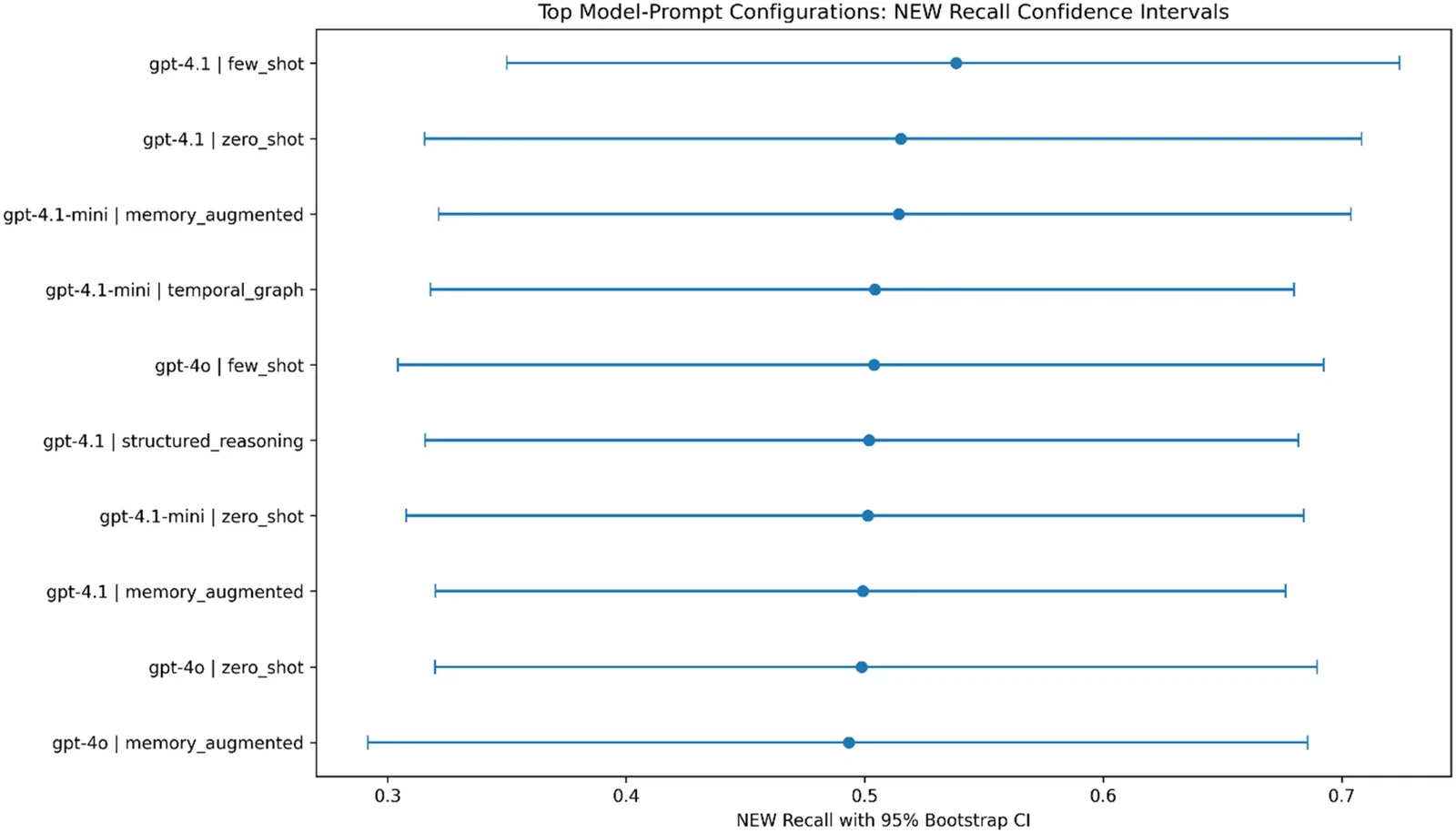
Bootstrap confidence intervals for new recall.

Table 10 presents bootstrap confidence intervals, and Figure 4 provides a visual comparison of the uncertainty in new class recall values within the model-prompt structure. This graph shows the point estimates and 95% bootstrap confidence intervals for new class recall of the best performing model-prompt combinations. For new class recall, the GPT-4.1—few-shot combination achieved the best performance with 0.5384. The confidence interval for this combination was determined as 0.3500–0.7420. The wide confidence interval indicates the sensitivity of performance in this class to sampling variability. This is related to the fact that this class is clinically more difficult to distinguish. It is observed that the GPT-4.1-mini—memory-augmented and GPT-4.1-mini—temporal graph combinations yielded good results for new class recall. However, combinations based on the GPT-4.1 model performed better for the macro-F1 metric. This shows that smaller models produce competitive results in certain classes, but the larger model is generally more advantageous.

## 6 Discussion

Among the configurations created for the study, the GPT-4.1 model provided the strongest and most consistent temporal reasoning. In this model, the best results were obtained with the few-shot and zero-shot configurations, with macro-F1 values of 0.789 and 0.787, respectively. The success achieved depends on both the model and the configuration. The same model yielded different macro-F1 scores with different configurations. Simpler configurations, such as zero-shot and few-shot, competed with and generally performed better than more complex memory-based and temporal graph configurations. This suggests that complex configurations may weaken the decision and not contribute to the pairwise temporal comparison of two observations. The GPT-4.1 model family is concentrated in less frequent and clinically more challenging classes. This is more evident in the model's priority in the macro-F1 metric rather than accuracy. Error cases are grouped into two points: missed novel findings and direction of change error. This group includes cases where novel findings are often misclassified as stable and resolved findings as improved. This is because these pairwise classes are semantically close to each other. The model's configuration, which forces it to check whether the current state is new or unchanged, has led to increased new-class recall in some small models. This is consistent with the error pattern.

Current studies in LLM related to radiology reports generally focus on information extraction, structuring, and summarizing from individual reports. Some of these studies have focused on temporal relationship inference in the event-timeline context. The findings obtained in this study are consistent with previous studies for majority classes. However, converting temporal relationships into a clinical decision label and identifying new findings is a limiting factor.

The new-class recall metric is at a moderate level in the best-configured model. This indicates that models should be positioned not as sole decision-makers but as auxiliary second-readers. In integration studies, reporting of possible new or worsening findings in the current study or prioritizing the worklist should be brought to the attention of radiology professionals. Since models highlight temporal changes, they can support decision support and follow-up planning. They can also be used in quality assurance and retrospective audit processes. The high level of precision achieved for new-class models makes them meaningful because they prevent the generation of numerous false alarms and detect inadequately reported intermediate changes. The most dominant error type is the most clinically critical. Failure to detect a new pneumonia, effusion, or consolidation can delay follow-up and treatment. Errors in the direction of change carry a similar risk. This indicates that the outputs of the models should be used as a guide, and expert evaluation should be required for final decision-making. Since a missed finding can be more costly than a falsely generated alarm, the working point should be adjusted to increase new-class recall. Furthermore, not only accuracy but also the types of errors that occur should be monitored in the working environment.

## 7 Conclusion

This study evaluated temporal novelty detection in chest radiography reports using LLM. The main objective of the study was to determine the temporal status of specific findings between a patient's current and previous reports and to compare their performance on this task using model-prompt configuration. For this study, zero-shot, few-shot, structured reasoning, memory-augmented, and temporal graph prompt strategies from the OpenAI, Anthropic Claude, and Google Gemini model families were used. This indicates that the study was not only based on model comparison but also examined the effect of prompt strategy on temporal clinical reasoning.

Experimental studies have shown that GPT-4.1 model-based configurations generally exhibit the best performance. Specifically, the combination of GPT-4.1 and few-shot prompts delivered the best performance. This combination produced strong and balanced results in terms of accuracy, macro-F1, weighted-F1, and new class recall values.

One of the key findings of this study is that model performance cannot be explained solely by accuracy. In clinically important tasks such as temporal novelty detection, macro-F1 and new class recall values better reflect the model's potential. Avoiding the loss of newly emerging findings is critical for clinical follow-up and decision support processes.

While bootstrap confidence intervals show similar results among configurations with good model-prompt performance, these differences are noteworthy. Confidence intervals partially overlap among prompt configurations of the high-performing GPT-4.1 model. This indicates that model-prompt combinations, rather than any single configuration being absolutely superior, achieve high performance. Paired McNemar significance testing confirmed that: the leading GPT-4.1 configurations are statistically indistinguishable (all *p* ≈ 1.0). This result suggests that score-based comparisons alone are insufficient for comparisons with LLMs; assessments incorporating uncertainty analysis provide more reliable interpretations.

The original contribution of this study is the combined evaluation of LLM with the LUNGUAGE dataset, not only in terms of overall classification success, but also in terms of temporal reasoning, prompt strategy sensitivity, error modes, novel finding detection, and statistical uncertainty. This provides a comprehensive evaluation framework for the use of LLM in radiology reports. The results show that LLM is powerful in interpreting temporal variation among radiology reports, but overall success values alone are insufficient for clinical reliability.

The study has some limitations. It was conducted using specific examples and label structures from the LUNGUAGE dataset. In addition, the comparative evaluation was performed on a class-stratified subset (*n* = 1,500) of the labelled pool. The results may vary in datasets containing different radiology reports or in different clinical settings. Model responses were evaluated using automated label normalizations. Further radiologist-assisted error review could be performed for more detailed clinical validation.

Future studies could improve the application of the evaluation framework to a larger sample size. This data could also be expanded with different radiology models and supported by the combined evaluation of image and textual data. As an alternative to closed-source models, the task in this study could be accomplished using open-source LLM with clinically specific fine-tuning methods and Retrieval-Augmented Generation-based approaches. For the critical new class, methods that more strongly structure finding matching, anatomical position normalization, and previous report context could be developed to reduce errors.

## Data Availability

The LUNGUAGE dataset used in this study is publicly available on PhysioNet (https://physionet.org/content/lunguage/1.0.0/). The original contributions presented in the study are included in the article/Supplementary Material. Further inquiries can be directed to the corresponding author.
